# Supplementary material for: Are carbon emissions trading and green financial instruments synergistic? -Comprehensive quantitative research based on content analysis
Source: PLoS One. 2024 Mar 7;19(3):e0298601. doi: 10.1371/journal.pone.0298601 (PMC10919601; doi:10.1371/journal.pone.0298601)
Supplement: S1 Data — (ZIP) [file pone.0298601.s001.zip › Data/地级市Z_F工作报告.zip!/╜¡╬≈/╔╧╚─╩╨/└┤╫╘╣1⁄2╓┌║┼í╛┬φ┐╦╩2╛▌═°í┐_2016.docx]

各位代表：

现在，我代表市人民政府，向大会报告政府工作，请予审议，并请市政协委员和列席会议的同志提出意见。

一、2015年工作回顾和“十二五”时期主要成效

过去一年，在市委坚强领导和市人大、市政协监督支持下，全市上下认真贯彻党的十八大、十八届三中、四中、五中全会和习近平总书记系列重要讲话精神，坚决落实“发展升级、小康提速、绿色崛起、实干兴赣”十六字方针，适应新常态，抢抓新机遇，统筹做好稳增长、促改革、调结构、优生态、惠民生各项工作，实现了经济社会平稳较快发展。全年生产总值1650.8亿元，增长9.5%;完成财政总收入290.1亿元，增长10.5%;规模以上工业增加值690.5亿元，增长9.6%;固定资产投资1560.2亿元，增长16.1%;社会消费品零售总额637.2亿元，增长12.7%，完成了市三届人大五次会议确定的主要目标任务。

(一)抓产业升级，发展效益稳步提升

“主攻工业”成效显著。强化工业支柱意识，加强园区建设，上饶经开区、广丰经开区主营业务收入分别突破700亿元、500亿元;加快产业集群发展，光伏产业销售收入占全省一半以上，晶科能源主营业务收入突破300亿元，成为全球领先的光伏企业，汉腾汽车成功下线，发动机项目顺利推进，以汽车为核心的先进制造业崭露头角;推进项目建设，建立市县两级 “工业日”制度，每季度均举行全市性工业项目集中开(竣)工活动，累计开竣工项目585个，广丰卷烟厂易地技改、弋阳海螺水泥扩产、玉山众光照明、德兴翔鹰航空零部件等重点项目进展顺利。工业用电量增幅列全省第3位，预计全市工业投资增幅列全省第2位、新增规模以上工业企业190户。

旅游产业发展迅猛。把旅游产业作为绿色崛起的先锋产业，大力开展全国旅游标准化试点工作，全年接待游客9344.4万人次，旅游综合收入790.3亿元，门票收入14.5亿元，旅游从业人员超过20万。三清山创建江西旅游示范景区初见成效，鄱阳湖国家湿地公园成功创评国家级生态旅游示范区，龟峰通过 5A级景区资源评审，新增灵山、熹园2个4A级景区，旅游品牌总量全省第一;组建上饶旅游发展集团，搭建了旅游资源整合的新平台;“厕所革命”先锋行动圆满完成全年任务;干细胞健康旅游等项目加快推进，《梦里老家》大型山水实景演出成功上演， “金驹杯”世界大学生摄影展如期开展，中华龙舟大赛顺利举办，与省移动公司合作的“会旅游”平台建成上线，“旅游+医疗养生”“旅游+文化产业”“旅游+体育产业”“旅游+互联网”等“旅游+”战略稳步实施;承办全省旅游产业发展大会，牵头成立沪昆、京福高铁旅游城市联盟，旅游宣传营销有声有色，区域旅游影响力、竞争力显著提升。

现代农业快速发展。预计全年粮食种植面积892.9万亩，总产352万吨，实现“十二连增”。成功创建万年国家级现代农业示范区和8个省级现代农业示范区，建成各类农业园区47个，核心区面积5.2万亩。新增市级以上农业龙头企业58家、家庭农场852家、农民专业合作社1613家，市级以上农业龙头企业实现销售收入300亿元，带动农户123万户，促进农民增收30亿元，农业产业发展“1+2+3>6”的综合效应逐步显现。

金融、科技、商贸、物流、电商等现代服务业健康发展。设立总规模10亿元的中小微企业转贷互助帮扶基金，远泉林业、艾芬达卫浴、巨网科技、耐普矿机、同欣机械制造等企业在“新三板”挂牌，银行业金融机构人民币存款余额2258.4亿元、增长18.5%，贷款余额1474.3亿元、增长19.5%，保险业实现保费收入48.3亿元、增长31.8%，赔款支付11.3亿元、增长34.1%。重点推进创新平台建设，成功申报了9个省级工程技术研究中心项目，新建1个省级科技孵化器;全年专利申请量2750件，增长90%。积极推进万达广场、星河国际等大型商业综合体建设，中心区商圈即将形成。规模以上物流企业发展至80家，其中4A级物流企业3家。全市电子商务经营主体达1.7万家，电子商务交易额突破300亿元。

(二)抓深化改革，发展动力持续增强

新一轮政府机构改革基本完成。加快“三单一网”改革，上网公布市、县政府权力清单和责任清单，公布两批负面清单。深化商事制度改革，全面实行“三证合一、一照一码”企业登记制度，全年新增私营企业近1万户，平均每天新增27户，新增个体工商户2.8万户，平均每天新增78户，创新创业活力初步显现。机关事业单位养老保险制度改革有序推进。不动产登记工作平稳实施。农村土地承包经营权确权登记颁证基本完成。第三批8个县级公立医院综合改革全面启动。强力推进投融资平台改革，成功组建上饶投资控股集团，融资额达146.4亿元，为投资兴业奠定了坚实资本基础。

(三)抓项目建设，投资关键作用充分体现

2015年，预计全市投资拉动经济增长4.7个百分点。推进了一批重大交通、能源、水利项目。德上高速怀玉山和大茅山连接线顺利竣工，上万高速全面施工，九景衢铁路建设快速推进。国省道改建350公里、农村公路硬化1500公里。开工建设上饶东、德兴两个500千伏变电站，全市电网最大负荷达181万千瓦。启动城东防洪堤等重点水利项目，除险加固一般小(2)型病险水库426座。加大产业投资、引资力度。预计完成产业投资1097.7亿元，增长 23.3%，占固投比重达70.4%，较上年提高4.1个百分点。新签约的382个项目中，工业项目262个，10亿元以上项目54个。围绕晶科能源引进 14个太阳能光伏发电项目，装机总容量超过1000兆瓦;围绕凤凰光学引进20家上下游光学企业;围绕汉腾汽车引进13个汽车产业配套项目。全年利用省外5000万元以上项目资金509.2亿元，增长16.2%;利用外资9.4亿美元，增长12.2%;外贸出口38.2亿美元，增长5.8%。积极争取国家和省级重大项目、专项基金。通过多方努力，高铁动车组存车场、坑口铁路专用线(货场)、上饶发电厂、鄱阳至余干高速等重大项目基本落实;全年争取国家专项建设基金投资项目60个、引导性基金26.6亿元;向省发改委申报了9大类1030个重大项目，并从中筛选了100个项目由市委、市政府直接调度，全年完成投资359.2亿元，为促进全市经济社会发展提供了有力支撑。

(四)抓城乡协调，新型城镇化步伐明显加快

中心城区建设力度加大。完成《上饶市城市总体规划》纲要论证及配套规划编制工作。加快推进上饶高铁站城市交通综合枢纽、老火车站片区综合改造、广信大厦等55个中心城区重点项目。推进上饶大道、天佑大道、吴楚大道、凤凰东大道延伸段等项目，着手打通城市“断头路”。加大控违拆违力度，遏制了中心城区违法建设蔓延的势头。

城乡建设有序推进。积极推进信江河谷城镇群和经济带建设，完成广丰撤县设区工作，改变了上饶市单一辖区历史;加快推进上铅、上广快速通道建设，初步构建了信江河谷城镇群“半小时经济圈”。鄱余万地区城镇建设步伐加快，三县的县城建成区面积共计60.6平方公里，较上年扩大4平方公里。1411个新农村建设任务全面完成。城乡环境卫生整治深入推进，农村生活垃圾集中处理率超过80%。

县域经济健康发展。广丰家具制造产业集聚效应不断增强，玉山装备制造产业开始集聚，万年电子信息产业快速发展，余干水钻产业初具规模，德兴遮阳产业发展迈出新步，铅山新能源产业发展迅速，信州商贸流通产业日益活跃，鄱阳五金机电产业扬优乘势，上饶县铜产业保持稳定，婺源旅游产业转型升级，弋阳水泥产业效益稳定，横峰物流产业势头良好。全市有8个县(市、区)的GDP超过100亿元;5个县(市、区)的财政收入超过20亿元，其中德兴超过30亿元，广丰突破40亿元。

(五)抓环境治理，生态文明建设力度加大

坚持铁腕治理生态环境，加大生态文明建设力度，全市河流断面水质达标率为96.6%，饮用水源达标率为100%，上饶市城区环境空气质量优良率为 94.2%。造林绿化28.6万亩，完成省下达任务的106%。国家森林城市创建工作扎实推进，并确定樟树为市树、三清杜鹃和山茶花为市花。重点推进了再生资源循环经济产业园、中心城区垃圾焚烧发电、城镇生活污水处理厂配套管网及县(市)工业园区污水处理厂建设。列入减排计划的169个项目全部建成投运。有效开展沪昆、京福高铁沿线环境整治工作，倾力打造 “最美高铁线”。

(六)抓民生保障，社会事业协调发展

民生事业不断加强。全市开展创业培训1.5万人，新增城镇就业6.2万人，新增转移农村劳动力19.5万人，脱贫13.7万人。城乡居民人均可支配收入分别达26924元、10112元，增长9.2%、11.1%。在全省率先启动“银发无忧”老年人意外伤害保险公益工程。创新出台《特困群体基本养老保险助保贷款暂行办法》。省政府下达我市保障性安居工程的开工率和基本建成率均达100%，政策性农房保险参保率达100%。加强信息惠民工作，成为全省第一个同时开展国家信息惠民试点城市、国家宽带中国示范城市、国家智慧城市试点县、国家信息消费试点县等试点工作的设区市。

社会事业全面进步。深入推进“十大整治”活动，启动了全国文明城市创建工作。优先发展教育事业，新改(扩)建校舍78.9万平方米。加强文化建设，万年稻作文化作为中国展示项目中唯一的江西元素亮相米兰世博会，江西矿冶博物馆在德兴开馆。全民健身和竞技体育蓬勃发展，成功举办了首届中式台球世锦赛、全国半程马拉松赛等大型赛事。大力推进卫生基础设施建设，启动了新人民医院、市卫生监督综合楼等重点医疗卫生项目。统计审计、民族宗教、涉台事务、人民防空、防震减灾、气象水文、检验检疫、妇女儿童、新闻出版、社科研究、住房公积金、无线电管理、红十字、档案、保密、石油、烟草、盐业、慈善等各项事业取得新成绩。

社会保持和谐稳定。全面推进应急管理体系建设和防灾减灾工作，安全生产形势平稳。坚持用群众工作统揽信访工作，健全了信访维稳长效机制。第九届村 (居)委会换届选举工作顺利完成，全省首家市级社会组织孵化基地正式挂牌。打击防范犯罪成效明显，严重影响群众安全感的“抢劫”“抢夺” “八类”严重暴力犯罪发案率同比分别下降36.9%、21.4%和20.4%，现行命案全破。

抓好经济社会发展各项工作，加强政府自身建设至关重要。一年来，市政府严格按照法定权限和程序行使权力、履行职责，自觉接受市人大及其常委会的法律监督、工作监督和市政协的民主监督，主动向人大常委会报告工作，认真接受人大代表询问和政协委员意见建议，按期办复人大代表建议66件、政协委员提案156 件，办结率和满意率均为100%;新建行政服务办事大厅，完成市政府网站改版，开通 “手机版”市长热线;普遍建立政府法律顾问制度，成为我省第一批获得地方立法权的设区市，法治政府、服务型政府建设展现新气象。

刚刚过去的一年，是“十二五”规划的收官之年。经过全市上下的共同努力，“十二五”上饶经济社会发展取得了重大成就，为全面建成小康社会打下了坚实基础。

这五年是综合实力显著提升的五年。“十二五”期间，预计生产总值年均增长10.8%，财政总收入年均增长20.6%，规模以上工业增加值年均增长14.0%，固定资产投资年均增长18.9%，社会消费品零售总额年均增长13.4%。

这五年是交通格局历史性跃升的五年。沪昆、京福高铁运行，三清山机场基本建成，上武、德昌、德上高速通车，上饶正式迈入高铁时代，即将结束没有民航交通的历史，立体式交通枢纽格局基本形成。

这五年是工业经济快速突破的五年。全市工业税收88.2亿元，是“十一五”末的1.7倍。预计新增工业园区面积30.7平方公里，实际开发面积94.5平方公里，列全省第1位;工业园区主营业务收入2650亿元，是“十一五”末的1.3倍。

这五年是“三农”工作扎实推进的五年。粮食连年增产并突破70亿斤，农村居民人均可支配收入突破万元大关，较“十一五”末增加4611元。五年累计筹措资金56亿元，建设新农村7529个，惠及村民44.2万户。

这五年是服务业迅猛发展的五年。全市服务业增加值达624.6亿元，实现五年翻番。三清山、江湾跻身国家5A级景区，全市旅游接待人数、旅游综合收入年均分别增长37.3%、41.5%，旅游成为上饶最靓的一张名片。金融总量达3732亿元，是“十一五”末的2.4倍。

这五年是城镇化驶入“快车道”的五年。城镇化率由39.7%提高到47.1%，年均提高1.5个百分点。荣获了中国优秀旅游城市、中国十大幸福城市、全国双拥模范城市、国家园林城市等国家级城市名片，上饶美誉度不断提升。

这五年是重点领域改革卓有成效的五年。深入贯彻中央和省里关于深化改革的重要部署，切实推进政府职能转变，精简市本级行政审批事项230项，承接国务院、省政府下放的审批事项154项;完成七个系统97户国企改革，安置职工12.5万人。

这五年是开放合作亮点纷呈的五年。赣东北扩大开放合作、上饶高铁经济试验区上升为省级战略。上饶—宁波“海铁联运天天班”成为我省一条快速绿色出海通道。五年累计利用省外5000万元以上项目资金1871.4亿元、年均增长21.8%，利用外资38.1亿美元、年均增长13.3%，外贸出口155.1 亿美元、年均增长20.2%。

这五年是生态优势巩固提升的五年。累计造林179.2万亩，林木绿化率达61.8%，较“十一五”末提高0.1个百分点。全市国家级森林公园、湿地公园、自然保护区、水利风景区数量位居全省前列。

这五年是民生保障稳步提高的五年。五年间城镇登记失业率控制在4.5%以内。城乡居民养老保险实现全覆盖。全面建立居民大病保险制度并实现市级统筹。社会救助体系基本建立，共发放城乡低保补助资金34.6亿元。累计脱贫30.7万人，贫困发生率由13.8%下降到6.1%。

各位代表，过去五年的成绩来之不易，这是认真贯彻党中央、国务院一系列重大决策部署的结果，是省委、省政府和市委正确领导的结果，是全市干部群众主动担当、共同拼搏的结果。在此，我代表市人民政府，向全市广大工人、农民、知识分子、干部和历任老领导、老同志，向各民主党派、工商联、无党派和社会各界人士，向驻饶人民解放军、武警官兵和公安民警，向中央和省驻饶单位及所有关心、支持上饶发展的同志们、朋友们，向在我市创业的投资者、建设者，致以崇高的敬意和衷心的感谢!

回顾过往，我们也清醒地看到，我市经济社会发展还存在很多不足。工业总量不大、结构不优，仍是我市经济均衡健康发展最大的短板;中心城区“首位度”不高，辐射力和带动力需进一步加强;旅游产业链条不长，综合带动效应发挥不够;招商引资的大项目、好项目不多，开放型经济仍处于较低水平;民生保障能力不足，财政收支矛盾突出;少数政府工作人员的思想仍然不够解放，与沿海发达地区的 “服务落差”依然存在。总而言之，发展不足、发展不充分，仍然是上饶最大的市情、最大的实际和最大的矛盾，我们必须牢固树立发展中心意识，通过发展的办法，解决前进中的问题。二、“十三五”时期的奋斗目标和战略任务

各位代表，“十三五”时期，上饶和全国一样，仍处于大有作为的重大战略机遇期。我们要进一步增强忧患意识、机遇意识、责任意识，保持定力，奋发有为，真抓实干，奋力开拓上饶发展新境界。

市委三届十次全会提出了《中共上饶市委关于制定全市国民经济和社会发展第十三个五年规划的建议》。市政府按照《建议》提出的工作重点，制定了《上饶市国民经济和社会发展第十三个五年规划纲要(草案)》，提交本次大会审议。

《纲要(草案)》提出了今后五年全市经济社会发展的指导思想，即：高举中国特色社会主义伟大旗帜，以马克思列宁主义、毛泽东思想、邓小平理论、“三个代表”重要思想、科学发展观为指导，深入贯彻习近平总书记系列重要讲话精神，遵循全面建成小康社会、全面深化改革、全面依法治国、全面从严治党的战略布局和创新、协调、绿色、开放、共享的发展理念，坚持发展升级、小康提速、绿色崛起、实干兴赣的“十六字”方针，紧紧围绕与全省全国同步全面建成小康社会的总目标，以扩量、提质、增效为中心，协同推进新型工业化、城镇化、信息化、农业现代化和绿色化，加快建设四省交界区域中心城市、江西绿色崛起副中心城市，全面打造富裕上饶、美丽上饶、人文上饶、幸福上饶。

《纲要(草案)》提出全市“十三五”时期经济社会发展的主要目标是：确保地区生产总值和城乡居民人均收入与2010年相比提前两年实现翻番，确保到 “十三五”期末人均地区生产总值突破6000美元、城乡居民人均收入达到全省平均水平，全面建成小康社会，为第二个百年目标奠定坚实基础。这些目标的确定，既考虑了与国家和省里“十三五”经济社会发展主要目标的衔接，确保上饶同步全面小康目标实现，又兼顾了上饶市情实际和特色优势，综合分析了上饶经济增长的潜力、发展的惯性、内外发展的环境。应该说，目标的设定，既鼓舞人心，又切实可行。

围绕上述目标，着眼适应和引领经济发展新常态，推进全市经济持续健康发展，《纲要(草案)》对我市国民经济和社会发展作出了全面部署，提出了十个方面的战略任务：一是均衡有序，优化空间发展格局;二是转型升级，推动产业发展迈向中高端;三是统筹发展，构建城乡一体新格局;四是互联互通，增强基础设施支撑能力;五是低碳环保，全力争当生态文明标兵;六是共享包容，全面增进人民福祉;七是开放合作，打造江西双向开放“桥头堡”;八是创新创业，提升经济发展质量和效益;九是深化改革，增添经济社会发展活力;十是依法治市，建设法治上饶平安上饶。制定这些战略任务，主要考虑有三个方面：一是全面贯彻中央精神和省里部署要求，十八届五中全会提出的“十三五”发展要求和中央经济工作会议提出的推动供给侧结构性改革、“三去一降一补”等战略部署，以及省里关于与全国同步全面建成小康社会的七大战略，都体现在了工作任务中。二是完整体现市委“规划建议”，是市委“1234”战略思路和发展举措的具体化。三是突出工作的连续性、针对性和可操作性，确保提出的战略任务能够顺应发展趋势、引领发展方向。三、2016年主要任务

2016年是实施 “十三五”规划的开局之年，是全面建成小康社会决胜阶段的第一年。总体看，今年经济下行的压力仍将持续，面临的发展形势更为严峻。我们要坚定信心，进一步强化发展中心意识、工业支柱意识、中心城区龙头意识、开放合作意识和创新创业意识，千方百计化挑战为机遇、化危机为生机、化被动为主动。

全市经济社会发展主要预期目标是：生产总值增长9%，财政总收入增长9%，一般公共预算收入增长9.5%，规模以上工业增加值增长9%，固定资产投资增长15.5%，社会消费品零售总额增长11.5%，外贸出口总额增长2%，实际利用外资增长10%，城镇居民人均可支配收入增长9%，农村居民人均可支配收入增长10.5%，居民消费价格总水平涨幅控制在3%左右，城镇登记失业率控制在4.5%以内，节能减排完成省里下达的计划任务。

重点抓好九个方面的工作：

(一)坚持主攻工业，加快工业产业升级步伐

坚定抓好工业的信心和决心，科学制定实施“工业翻番”计划，努力打造全省重要的先进制造业基地。

加快平台建设。继续创建高新技术园区，加快建设智能化和生态化园区，推动园区升级。加快省级工业园区扩区调区步伐，力争年内5个园区获省政府批准。支持上饶经开区做大做强，确保全年主营业务收入超过800亿元。

做大产业集群。建立龙头企业“直通车”制度，下大力气培育一批拥有自主核心技术、辐射面广、抗风险强的大型企业集团。发挥龙头企业带动作用，推进晶科能源四期、展宇新能源三期等项目，创建国家新能源示范城市。抓好光学产业，促进凤凰光学快速发展。加快推进汉腾汽车整车生产扩能和汽车发动机、彩诚实业集团汽车零部件等项目，将汽车产业培植成为我市新的支柱产业。

优化产业结构。实施新一轮传统产业技术改造升级工程，重点支持有色金属、建材等传统产业提质增效。对接“中国制造2025”战略，深入实施“互联网+”行动计划，促进新一代信息技术与制造业融合，打造智能工厂和数字化车间。

积极帮扶企业。大力开展降低实体经济企业成本行动，全面降低企业交易、财务、物流成本，降低税费、社会保险费负担，降低电力价格，让企业有更多的资金用于投资、创新、转型。全面提高服务质量，切实帮助企业搭平台、拓市场、争订单、解难题，促进企业健康发展。

(二)坚持高点定位，建设全国旅游强市

充分发挥上饶交通便捷、旅游资源密集的优势，进一步加快旅游业发展，力争全年旅游人数、门票、综合收入、从业人数分别增长20%以上。

全面提升旅游接待能力。围绕“吃、住、行、游、购、娱”等环节做全旅游要素，积极推进上饶旅游集散中心、三清山中国道教文化园、大灵山禅文化园、婺源耐克体育运动健身度假区、葛仙山景区升级改造及索道建设、大茅山索道栈道游步道、怀玉山景区、铜钹山白花岩景区、大明湖景区、鄱阳湖大草原体验区等十大旅游项目，进一步丰富旅游业态，创新旅游产品;加强配套设施建设，进一步完善旅游交通体系;探索组建“旅游警察”队伍，提高旅游管理服务水平，为把上饶建设成为华东地区重要的旅游节点城市、全省最大旅游集散地打下坚实基础。

构建全域旅游新格局。积极推动铜钹山、葛源、葛仙山、云碧峰、广丰红木文化创意产业园、玉山金沙溪梦里水乡创建4A级景区;启动篁岭、鄱阳湖国家湿地公园创建5A级景区工作，确保龟峰成功创建5A级景区。发挥上旅集团作用，完成部分优质旅游资源整合。高品位、高品质打造城市旅游主产品，力促灵山景区客流量大幅增长，三宝街、石狮坊等项目快速推进，将中心城区建成游客必到、必看、必游、必购的旅游目的地。科学论证和规划三清山—德兴(港首)—婺源生态旅游先导区建设项目。大力发展休闲农业、乡村旅游和民宿经济，加快旅游扶贫步伐，努力形成“景城呼应、城乡并举”的全域旅游发展新格局。

深化“旅游+”发展战略。制定大健康旅游产业发展规划，推进上饶(国际)干细胞再生医学产学研基地建设，申报省级乃至国家级医疗旅游先行区。继续推进智慧旅游建设，拓展“会旅游”平台综合服务功能。加大非物质文化遗产进景区力度，加快旅游演出项目建设，提升上饶旅游产品的文化内涵。

加强旅游开放合作。全面参与赣浙闽皖四省旅游合作，携手创建“国家东部生态旅游实验区”。强化高铁沿线旅游宣传营销，做热高铁旅游市场。深化与港澳台“一程多站”的旅游合作，开拓以韩国、俄罗斯及东南亚为主要目标的国外市场，扩大上饶旅游国际影响力。

(三)坚持融合发展，提升农业现代化水平

以农民增收为目标，以农业龙头企业为依托，以转变农业发展方式为动力，加快建设高端的绿色生态农副产品供应基地。

大力发展现代农业。开展粮食高产创建行动，粮食种植面积稳定在890万亩左右。积极推进农村一二三产业融合发展，大力实施“十百千万”工程，打造现代农业生态体验区。加快发展粮食、水产、蔬菜(葛)、生猪(畜禽)、茶叶、水果、油茶、毛竹、苗木(花卉)、药材等特色产业，不断提高上饶农产品的市场占有率。

推进农业适度规模经营。引导农业要素向优势经营主体集中，力争市级以上农业龙头企业销售收入达320亿元，新增家庭农场200家、农民专业合作社 300家。强化重大动植物疫病防控和农产品质量安全监管，确保“餐桌上的安全”。不断拓展农业功能，提升农业的休闲、文化、生态价值，带动农民增收致富。

加快美丽乡村建设。优化乡村布局，抓好1400个左右新农村村点建设。规范农村建房管理，下大力气解决农民建房杂乱无序问题。差别化推进村庄整治和设施建设，努力打造 “六好”秀美乡村。

(四)坚持繁荣三产，做大做强现代服务业

大力实施服务业提速工程，不断创新服务业发展模式和业态，打造“上饶服务”品牌。

加强金融工作。力争全面完成农商行改制工作，努力实现村镇银行县域全覆盖。探索建立资本市场服务中心、民间借贷登记服务中心和“互联网+”金融服务中心;争取与国内知名的基金公司、证券公司、投资公司合作，设立股权投资基金。推进中小企业信用体系建设，严厉打击恶意逃废债行为和非法集资活动。构建政保合作平台，全面扩大保险服务领域。力争全年新增银行业金融机构人民币存款315亿元、贷款245亿元、资本市场直接融资80亿元以上，保险业保费收入同比增加 12.5亿元，新增各类股权交易市场挂牌企业10家以上。

抢先发展大数据产业。抓好与中科院战略合作协议的落实工作，联合中科院相关院所、企业，大力推进以大数据应用企业为主导的中科(上饶)创新产业园建设，努力将上饶打造成四省交界大数据枢纽和智慧型城市。

抓好商贸流通产业。瞄准国内外商贸流通行业龙头企业，力争引进一批能够积攒人气的大型市场、大型商场、大型酒店和大型物流企业。

加快电商产业发展。深入实施“电子商务进农村(社区)”工程，建设600个左右农村(社区)电子商务服务站。提升电商产业发展水平，打造一批具有全国影响力的电商企业和平台，着力创建国家电子商务示范城市。

大力发展文化创意产业。加强文化产业公共服务平台建设，加快发展文化旅游、影视娱乐、动漫网游等产业，着力打造一批有影响力的文化创意产业集聚区。

(五)坚持城乡统筹，大力推进新型城镇化建设

按照“精细优美”的理念，构建“一核两轴两板块”的总体开发格局，打造内陆城镇化建设示范区。

统筹抓好重大基础设施建设。完成三清山机场建设，争取尽早通航。支持九景衢铁路建设，积极推进坑口铁路专用线(货场)、动车组存车场、物流园等项目。加快建设老320国道全线升级改造等项目，完成上万高速、新320国道上饶城区段改建、上饶至广丰快速通道项目，开工建设鄱阳至余干高速，积极推进沪昆高速拓宽升级、昌景黄高铁、上饶至江山(廿八都)高速、信江高等级航道、鄱阳港等项目前期工作。开工建设上饶火力发电厂。确保上饶东、德兴两个500千伏变电站建成投运。加快推进天然气管网及加气站建设。扎实做好大坳灌区、夏营水库等项目前期工作，力争早日开工。加大“小山塘”整治及内涝治理。建设高标准农田40万亩。加快气象现代化建设，提升公共气象服务能力。推进粮食仓储设施建设，促进粮食转化利用和减损增效。

强化中心城区的核心地位。支持广丰区对接和融入中心城区，推进上饶县撤县设区工作，做大做强市本级实力，加大力度解决市民关心的就业、住房、教育、医疗、出行、购物等问题。抓好规划编制工作，完成《上饶市城市总体规划》编制并报省政府审批，开展《地下综合管廊工程建设规划》《上饶市排水防涝设施建设规划》以及高铁新区、空港新区、带湖老城区控规编制工作，加强重要地段、重要节点的城市设计。围绕宜居宜业宜游的目标，扎实推进市规划展示馆、博物馆、美术馆、群艺馆、档案馆、老年福利中心、妇女儿童活动中心、食品药品检验检测中心、新火车站综合枢纽立体停车场、三江导托渠等十大功能性项目，扎实推进上饶一中新校区、上饶三中、城东学校、第四幼儿园、新人民医院、儿科住院大楼、城东三甲医院、市精神卫生中心、市疾控中心综合实验楼、市卫生监督综合楼等十大教育卫生项目，扎实推进大数据中心、呼叫和服务外包中心、创新创业孵化中心、建筑科技产业园、饶商总部基地、高铁农都、大型现代农副产品交易中心、电竞城市综合体、互联网创新技术产业园、城东大型旅游综合体等十大高铁新区项目，扎实推进上饶大道、天佑大道、稼轩大道、吴楚大道、明淑大道、滨江东路、葛仙山路、槠溪路、龙潭路、庆丰北路等十大城市路网项目。推进城市地下综合管廊和海绵城市建设，提高城市防洪排涝减灾能力。推进上饶东、上饶西两个高速出口景观改造，提升上饶市区入口形象。加快户籍制度改革，有序推进农业转移人口市民化。打通供需渠道，消化库存，稳定房地产市场。狠抓控违拆违工作，坚决维护公平正义。加快数字城管指挥中心平台建设，推进市政、园林等作业市场化改革，持续开展城市环境卫生整治，逐步实现城市管理的网格化、智能化、精细化。

加快两大城镇发展轴建设。沿沪昆城镇发展轴，加快工业产业发展，打造 “产业合作、互利共赢、特色明显、竞争力强”的工业走廊。沿京福城镇发展轴，加快生态旅游产业发展，打造“山水秀美、生态宜居、城景交融、和谐发展”的黄金旅游线。

抓好东西两大板块建设。加快推进以信江河谷城镇群区域为核心、以德兴和婺源为延伸腹地的东部信江河谷板块，着力建设全省一流、极具上饶特色的森林城镇簇群。积极推进西部滨湖板块建设，加速融入南昌核心增长极，构筑我市西部发展的“金三角”。

(六)坚持改革创新，激发经济发展新活力

进一步加大扶持力度，推进创新型城市建设，努力将大众创业、万众创新培育成为加快经济发展的新引擎。

强力推进重点领域改革。深化“三单一网”改革，推进全市网上审批和协同办公应用，实现所有事项全流程、并联式网上公开审批。深化国资国企改革，稳步发展混合所有制经济，有序推进经营性国有资产集中统一监管。深化投融资改革，研究设立政府与社会资本合作(PPP)等投融资引导基金，吸引社会资本进入重大基础设施、社会事业和生态环保等领域。深化财税体制改革，建立全面规范、公开透明的现代预算制度，不断完善全市综合治税平台功能。深化城市管理和执法体制改革，改进和提升城市管理工作。深化农业农村改革，全面完成农村土地承包经营权确权登记颁证工作，继续抓好小型水利工程管理体制改革、水权改革、河湖管理体制改革、供销社综合改革、农垦改革、林业改革。与“滴滴出行”合作，利用互联网信息技术，做好公车改革及后续服务工作。

全力扶持大众创业。研究设立市级创业投资引导基金，引进天使投资、创业投资、风险投资，提高个体创业担保贷款额度和创业费用补贴标准。大力培育创客文化，支持中小微企业“二次创业”，支持农民工、大学生和退役士兵“返乡创业”。

大力实施创新工程。强化企业创新主体地位，鼓励企业与高校、科研院所建立科技创新协同中心，鼓励有条件的企业牵头制定国家标准、行业标准。加快推进国家级光学、新能源、汽车、干细胞、生物发酵食品添加剂等研发中心和产品检测中心建设。研究制定更加开放、更加灵活的人才政策，充分利用高铁优势，吸引大中城市高端人才来我市创业和服务。

(七)坚持开放战略，着力实现合作共赢

强化“掉头向东”的战略取向，努力提升我市对外开放合作水平，打造江西对外开放合作“桥头堡”。

积极对接融入国家战略。对接融入“一带一路”、长江经济带、长江中游城市群等国家战略，紧紧围绕我市经济发展需求，积极争取相关政策扶持，建设长三角和海西经济区重要腹地。运用第四届“万里茶道”与城市发展中蒙俄市长峰会的成果，积极开拓新兴市场。围绕打造中部地区重要的产业转移承接示范区的定位要求，抓紧谋划和建设跨省域重大发展平台。

继续提升招商引资热度。坚持把招商引资作为“一号工程”，着力引进世界和国内500强企业、跨国公司、行业龙头企业。瞄准优势产业、特色产业链条中的 “缺口”，大力开展产业招商，补齐产业“短板”，拉长产业链条。大张旗鼓地推进“饶商回归”工程，致力把“饶商”企业培育成为上饶新一轮发展的“生力军”。

切实做好外经外贸工作。加快推进光伏、茶叶、机电、特色农产品等优势产业出口基地建设，打响一批出口品牌。借助省级“走出去”融资统保平台，支持企业“走出去”，参与国际竞争合作。

大力促进口岸互联互通。推进上饶三清山机场口岸物流平台建设。结合上饶铁路货站西移，启动上饶“无水港”扩建工作。抓好上饶—宁波“海铁联运天天班”的揽货工作，保障“天天班”稳定运行。

(八)坚持生态立市，争当生态文明标兵

加强生态建设，不断放大“生态红利”和“绿色福利”，擦亮“大美上饶”品牌。

加强环境保护治理。全面推进“净水”“净空”“净土”行动，继续加快全市生活污水处理厂污水管网建设，加强大气和重金属污染防治，加快实施中心城区生活垃圾焚烧发电项目。全面落实“河长制”，加强河湖管理与保护。完成造林绿化24.4万亩，积极创建国家森林城市。落实生态补偿机制，完善资源有偿使用制度。继续推进环境保护大检查和清理违法违规建设项目，保持严厉惩处环境违法的高压态势。

推动绿色循环发展。继续推进再生资源循环经济产业园建设，积极争取将德兴市列入国家级“城市矿产”示范基地。抓好工业园区的循环化改造，力争上饶经开区成为省级“清洁化”工业园区。大力发展无公害农产品、绿色产品和地理标志农产品，构建生态有机的绿色农业体系。培育生态文化，提高生态意识，建设绿色家园。

(九)坚持民生优先，增强人民群众获得感

践行共享发展理念，加大民生投入，织牢民生兜底的“安全网”，不断增进人民群众福祉。

坚决打好脱贫攻坚战。坚持精准识别、精准结对、精准帮扶、精准管理、精准脱贫，综合运用产业扶贫、搬迁扶贫、生态补偿、保障扶贫、教育扶贫等各种扶贫方式，形成“单位帮扶、干部包户、社会参与、民政托底、结对帮扶”的精准扶贫新格局，促进105个贫困村摘帽，确保实现年度脱贫12.7万人的目标。

扎实推进民生工程。完善城乡均等的公共就业创业服务体系，提高就业服务的专业化、信息化水平。完善多层次社会保障，提高城乡低保、农村五保等保障水平。加大优抚力度，建设标准化救灾物资储备库。积极应对人口老龄化，加快养老服务体系建设，谋划好上饶市社区(村)居家养老服务中心项目。推进卫生信息化，加快人口健康信息云平台“26312”工程建设。继续抓好保障性安居工程，全面完成省政府下达的任务。

大力繁荣社会事业。科学调整中心城区各类学校整体布局，集中力量建设一批学校，努力化解“大班额”问题;大力发展职业教育，积极发展高等教育、特殊教育和学前教育。进一步抓好国家基本公共卫生服务均等化项目落实，继续推进村卫生室纳入乡镇卫生院一体化管理。稳步推进文化基础设施项目，实施农村智能广播网“村村响”工程。积极开展全民健身活动，办好各类体育赛事。加强关心下一代工作。认真执行全面放开二孩政策，提升优生优育服务水平。全面开展不动产统一登记工作。深入推进军民融合发展，加强国防动员和人民防空建设，切实做好双拥工作。加强防震减灾体系建设。重视发展慈善和社会公益事业。

不断创新社会治理。扎实推进平安上饶建设，严厉打击各类违法犯罪活动;织密“五张网”，构建全社会立体化大防控格局。加大安全生产监管力度，落实企业主体责任，坚决杜绝各类重特大事故发生。加强初信初访办理工作，推动部门工作会商，合力解决信访问题。完善突发事件应急管理机制，提升源头治理和应对处置水平。

各位代表!抢抓机遇，应对挑战，全面完成今年各项任务，需要政府切实加强自身建设。要进一步弘扬方志敏精神，学习先烈坚定的理想信念、伟大的爱国情怀、可贵的政治品格和崇高的气节风范，打造为民、务实、清廉、高效的政府系统干部队伍。要进一步增强法治意识，严格按照法定权限和程序履行政府职能，做到法定职责必须为、法无授权不可为，健全政务公开机制，切实保障人民群众的知情权、参与权、表达权和监督权。要进一步增强担当意识，以“三严三实”严格要求政府工作人员，引导广大干部把精力投在抓工作上、把本事用在抓发展上、把能力和水平体现在抓落实上，全心全意为企业服务、为基层服务、为群众服务。要进一步增强合作意识，树立全市上下“一盘棋”的思想，注重协调配合，搞好工作衔接，真正做到心往一处想、劲往一处使、拧成一股绳，共同把工作做好。要进一步增强廉政意识，持续加大“四风”整治力度，深入开展专项巡查，严肃查处发生在群众身边的各类腐败问题，始终保持惩治腐败的高压态势。

各位代表，上饶新一轮大建设、大提升、大发展的蓝图已经绘就。让我们紧密团结在以习近平同志为总书记的党中央周围，在省委、省政府和市委的坚强领导下，进一步解放思想、坚定信心、苦干实干，为建设四省交界区域中心城市、江西绿色崛起副中心城市而努力奋斗!
